# Hemostatic Effects of Exercise-related Hypoglycemia in Male Persons With Type 1 Diabetes

**DOI:** 10.1210/clinem/dgae278

**Published:** 2024-04-20

**Authors:** Per Gustav Hagelqvist, Andreas Andersen, Kaisar Maytham, Christine Rode Andreasen, Susanne Engberg, Ulrik Pedersen-Bjergaard, Julie Lyng Forman, Pär Johansson, Jens Lykkesfeldt, Filip Krag Knop, Tina Vilsbøll

**Affiliations:** Clinical Research, Steno Diabetes Center Copenhagen, University of Copenhagen, DK-2730 Herlev, Denmark; Center for Clinical Metabolic Research, Gentofte Hospital, University of Copenhagen, DK-2900 Hellerup, Denmark; Clinical Research, Steno Diabetes Center Copenhagen, University of Copenhagen, DK-2730 Herlev, Denmark; Center for Clinical Metabolic Research, Gentofte Hospital, University of Copenhagen, DK-2900 Hellerup, Denmark; Clinical Research, Steno Diabetes Center Copenhagen, University of Copenhagen, DK-2730 Herlev, Denmark; Center for Clinical Metabolic Research, Gentofte Hospital, University of Copenhagen, DK-2900 Hellerup, Denmark; Clinical Research, Steno Diabetes Center Copenhagen, University of Copenhagen, DK-2730 Herlev, Denmark; Center for Clinical Metabolic Research, Gentofte Hospital, University of Copenhagen, DK-2900 Hellerup, Denmark; Clinical Research, Steno Diabetes Center Copenhagen, University of Copenhagen, DK-2730 Herlev, Denmark; Department of Clinical Medicine, Faculty of Health and Medical Sciences, University of Copenhagen, DK-2200 Copenhagen, Denmark; Department of Endocrinology and Nephrology, Nordsjællands Hospital Hillerød, University of Copenhagen, DK-3400 Hillerød, Denmark; Section of Biostatistics, Department of PublicHealth, Faculty of Health and Medical Sciences, University of Copenhagen, DK-1014 Copenhagen, Denmark; Department of Clinical Medicine, Faculty of Health and Medical Sciences, University of Copenhagen, DK-2200 Copenhagen, Denmark; Clinical Academic Groups, Center for Endotheliomics, Rigshospitalet, University of Copenhagen, DK-2100 Copenhagen, Denmark; Section of Experimental Animal Models, Department of Veterinary and Animal Sciences, Faculty of Health and Medical Sciences, University of Copenhagen, DK-1870 Frederiksberg, Denmark; Clinical Research, Steno Diabetes Center Copenhagen, University of Copenhagen, DK-2730 Herlev, Denmark; Center for Clinical Metabolic Research, Gentofte Hospital, University of Copenhagen, DK-2900 Hellerup, Denmark; Department of Clinical Medicine, Faculty of Health and Medical Sciences, University of Copenhagen, DK-2200 Copenhagen, Denmark; Clinical Research, Steno Diabetes Center Copenhagen, University of Copenhagen, DK-2730 Herlev, Denmark; Center for Clinical Metabolic Research, Gentofte Hospital, University of Copenhagen, DK-2900 Hellerup, Denmark; Department of Clinical Medicine, Faculty of Health and Medical Sciences, University of Copenhagen, DK-2200 Copenhagen, Denmark

**Keywords:** cardiovascular disease, coagulation, fibrinolysis, hypoglycemia, type 1 diabetes

## Abstract

**Context:**

People with type 1 diabetes (T1D) are at increased risk of thrombosis compared to the general population; however, the underlying mechanisms remain unclear. Hypoglycemia induced at rest can induce coagulation activation, but little is known about the hemostatic effects of exercise-related hypoglycemia in people with T1D.

**Objective:**

We compared hemostatic profiles of individuals with T1D with healthy controls and explored hemostatic effects of hypoglycemia, induced with or without exercise, in participants with T1D.

**Methods:**

Thrombelastography was used for a baseline hemostatic comparison between fifteen men with T1D and matched healthy controls. In addition, the participants with T1D underwent two euglycemic-hypoglycemic clamp days in a randomized, crossover fashion. Hypoglycemia was induced with the participants at rest (Hypo-rest) or during exercise (Hypo-exercise). Thrombelastography provides data on the rate of coagulation activation (R-time), the rate of clot formation (K-time, α-Angle), the maximum clot amplitude (MA), the functional fibrinogen contribution to the clot strength (MA-FF) and the fibrinolysis (LY-30).

**Results:**

The T1D group exhibited a faster rate of coagulation activation (shorter R-time) and a faster clot formation (greater α-Angle) compared with the controls. During the clamp experiments, Hypo-exercise induced an increased clot strength (MA) with a mean difference from baseline of 2.77 mm (95% CI, 2.04-3.51) accompanied with a decreased fibrinolysis (LY-30) of −0.45 percentage point (−0.60 to −0.29). Hypo-rest resulted in increased functional fibrinogen (MA-FF) of 0.74 mm (0.13-1.36) along with an increased fibrinolysis (LY-30) of 0.54 percentage point (0.11-0.98).

**Conclusion:**

Individuals with T1D exhibit a hypercoagulable hemostatic profile compared with healthy controls and exercise-related hypoglycemia may increase the susceptibility to thrombosis via both procoagulant and antifibrinolytic effects.

People with type 1 diabetes (T1D) have a higher mortality rate compared with the general population and cardiovascular disease is 1 of the predominant causes of death in this group of individuals (1). T1D is a hypercoagulable condition with an increased risk of thrombosis, but the underlying mechanisms are not completely understood (2). Previous studies, based on conventional coagulation tests have shown that people with T1D, in comparison with healthy individuals, have increased levels of procoagulant factors (3, 4) and decreased levels of anticoagulant factors (5) and thus a potentially decreased threshold for thrombosis. Although plasma-based coagulation assays may provide details of specific coagulation factors, their capability to assess the initiation process of coagulation, the dynamics of clot strength, and to predict hypercoagulability is limited (6). A whole-blood global viscoelastic test (ie, thrombelastography [TEG]) can be a useful tool in stratifying risk of thrombosis (7). However, the use of TEG to assess the hemostatic balance in people with T1D is sparse (8, 9).

Intensive glycemic control can effectively reduce the development of microvascular complications in T1D (10), but it also increases the risk of hypoglycemia, which has been associated with increased cardiovascular mortality (11). The relation between hypoglycemia and cardiovascular disease is not fully understood but is most likely multifactorial and may include both proarrhythmogenic (12) and prothrombotic effects, including coagulation activation, endothelial dysfunction, and/or oxidative stress (13-15). Regarding the hemostatic system, previous experimental studies have focused on investigating the effects of hypoglycemia when induced under resting conditions (13, 15, 16). However, in real life, exercise-related hypoglycemia is frequently encountered in individuals treated with insulin (17), but a detailed description of its hemostatic consequences remains.

Here, we used a new-generation TEG, TEG 6s , to compare hemostatic profiles of young individuals with T1D with matched healthy controls and to delineate acute hemostatic effects of exercise-related hypoglycemia vs hypoglycemia induced at rest in individuals with T1D. In addition, we measured the sympathoadrenal response during and after hypoglycemia and explored the effects of hypoglycemia on markers of endothelial function and vascular oxidative stress.

## Materials and Methods

### Approval and Registration

The present study contains hemostatic data from a study investigating electrocardiographic changes during exercise-related hypoglycemia (18). Results obtained by TEG and other related secondary outcomes were by protocol planned to be published separately. The study was conducted in accordance with the Declaration of Helsinki and approved by the Scientific Ethical Committee of the Capital Region of Denmark (August 2020) (H-20023688) and the Danish Data Protection Agency (P-2020-343) and registered at ClinicalTrials.gov (NCT04650646). The study was carried out at Steno Diabetes Center Copenhagen, Herlev, Denmark, in cooperation with Center for Clinical Metabolic Research, Gentofte Hospital, Hellerup, Denmark. Oral and written consent was obtained from all participants before study inclusion.

### Study Design and Participants

**Figure 1.**
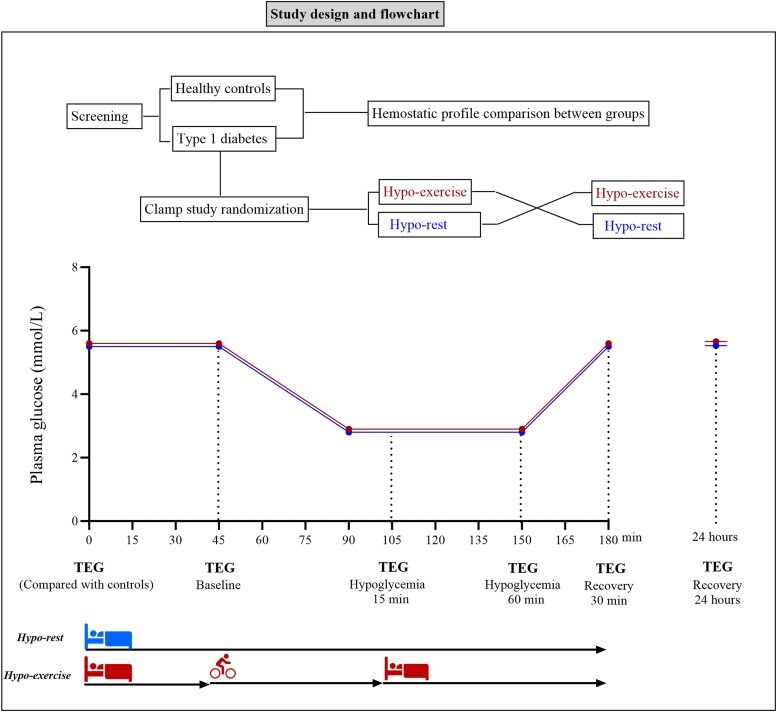
Study design and flow chart. Participants with T1D and healthy controls were screened before inclusion in the first part of the study—hemostatic profile comparison between groups. In the second part of the study, only participants with T1D were included and underwent 2 separate hyperinsulinemic hypoglycemic clamp days in a crossover randomized design. Hypoglycemia was induced along with a bout of exercise (Hypo-exercise) or with the participants at rest (Hypo-rest). TEG analysis was performed at baseline, after 15 minutes duration of hypoglycemia, after 60 minutes duration of hypoglycemia and after 30 and 24 hours of recovery. Hypo-rest represents hypoglycemia induced at rest and Hypo-exercise, represents exercise-related hypoglycemia.

Abbreviations: hypo, hypoglycemia; min, minutes; T1D, type 1 diabetes; TEG, thrombelastography.

Fifteen men with T1D and 15 healthy controls were included in the present study. Participants with T1D were recruited from the outpatient clinic at Steno Diabetes Center Copenhagen, whereas the healthy controls were recruited from a Danish clinical trial recruitment portal. The study consisted of 2 separate parts; first, a hemostatic comparison, after an overnight fast, between the group of individuals with T1D and the healthy controls, who were matched according to age, sex, and body mass index. Second, a hypoglycemic clamp study with hemostatic assessment during exercise-related hypoglycemia vs hypoglycemia at rest was performed (Fig. 1). The clamp study involved the participants with T1D only, and details of the clamp procedure have previously been reported (18). One test day was carried out entirely at rest, entitled “Hypo-rest,” whereas the other test day included moderate-intensity cycling exercise during the plasma glucose (PG) decline phase and the initial 15 minutes of hypoglycemia, entitled “Hypo-exercise” (Fig. 1). Blood samples for TEG, markers of endothelial activation and damage, and markers of vascular oxidative stress were collected during euglycemia (baseline), after 15 and 60 minutes duration of hypoglycemia and after 30 minutes and 24 hours of recovery.

### Plasma Glucose and Counterregulatory Hormonal Response

PG was measured every 5 minutes throughout the entire clamp procedure on both test days and in the fasting state after 24 hours. Plasma adrenaline and noradrenaline were measured at baseline, during hypoglycemia, and recovery on both test days. Details regarding the sampling procedure, timing and the analytical methods of PG, adrenaline, and noradrenaline have been described elsewhere (18).

### Hemostatic Assessment

TEG 6s (Haemonetics, Signy-Avenex, Switzerland) was used in both parts of this study. For the TEG analysis, whole blood was collected into a sodium citrate tube (3.2% trisodium citrate) and carefully inverted 5 times to ensure anticoagulation. All TEG analyses were made according to the manufacturer's recommendations (19). A whole blood sample from the sodium citrate tube was pipetted into a standard hemostasis assay cartridge containing four separate channels with calcium chloride together with the following dried reagents; (1) kaolin, (2) kaolin + tissue factor activated (rapid TEG), (3) kaolin + heparinase, and (4) kaolin + abciximab (functional fibrinogen). The following TEG parameters were used for statistical comparisons during both parts of the study: reaction time (R-time), representing the time of initiation of the coagulation cascade; kinetics (K-time) and α-Angle representing the rate of clot formation and fibrinogen polymerization; maximum amplitude (MA) representing the clot strength; MA-FF representing clot strength based on functional fibrinogen contribution; and finally, fibrinolysis (LY-30) representing the process of clot breakdown, measured as the percentage of clot lysis after 30 minutes. To compare hemostatic profiles between the participants with T1D and healthy controls, TEG was performed in the morning after an overnight fast. The timing of the TEG analyses in the clamp study is illustrated in Fig. 1.

### Endothelial Activation and Damage

For measurement of syndecan-1, platelet endothelial cell adhesion molecule 1 (PECAM-1), and thrombomodulin, whole blood was collected into EDTA tubes and centrifuged, and plasma was stored at −80 °C until analyzed with enzyme-linked immunosorbent assay (syndecan-1 [Catalog # 950.640.048, RRID:AB_3096170 Diaclone SAS, Besancon, France], PECAM-1 [Catalog # DCD310, RRID:AB_3096168, R&D System, Minneapolis, USA], and thrombomodulin [Catalog # 850.720.048, RRID:AB_3096166, Diaclone SAS, Besancon, France]). Markers of endothelial activation and damage were analyzed at similar time points as the TEG analyses.

### Vascular Oxidative Stress

Blood samples for measuring ascorbic acid and its oxidation product dehydroascorbic acid (DHAA) were immediately centrifuged, and then 1 aliquot of plasma was mixed and stabilized with 1 aliquot of meta-phosphoric acid solution (10% containing 2 mM EDTA). The plasma-meta-phosphoric acid solution mixture was further centrifuged, and the supernatant was stored at −80 °C until analyzed. The concentration of ascorbic acid (reduced form) and total ascorbic acid (ie, ascorbic acid + DHAA) were measured by HPLC coulometric detection (20). The fraction of DHAA (%DHAA) to the total ascorbic acid concentration, expressed in percentage, was used as a marker of oxidative stress (21). For measurement of malondialdehyde, whole blood was collected into EDTA tubes and after bedside centrifugation, plasma samples were stored at −80 °C until analyzed by HPLC. L-arginine and asymmetric dimethylarginine (ADMA) were analyzed by HPLC with fluorescence detection as reported elsewhere (22). Blood sampling for vascular oxidative stress analyses was performed at time 0 minutes (baseline), after 15 and 60 minutes of hypoglycemia, and after 30minutes and 24 hours of recovery.

### Statistical Analysis

The statistical analyses were carried out using SAS studio version 3.71 (SAS Institute Inc., Cary, North Carolina, USA). Data describing baseline demographics and components of the clamp procedure were summarized as mean ± SD, whereas skewed data were presented as median (interquartile range). Study endpoints were presented as mean with a 95% CI. Because of skewed distribution, the following parameters were logarithmically transformed before statistical comparison: K-time, thrombomodulin, syndecan-1, and plasma adrenaline. In the first part of the study, an unpaired *t*-test was used for statistical comparisons of TEG parameters between individuals with T1D and healthy controls. Equality of variances was tested and the Satterthwaite method used in case of unequal variances. In the second part, a linear mixed model was applied to evaluate changes within and between test days in TEG parameters, markers of endothelial activation and damage, and vascular oxidative stress. Time points, study visit (first vs second), test day (Hypo-rest vs Hypo-exercise), and the interaction between test day and time point were included as fixed effects and an unstructured covariance pattern was assumed to account for repeated measurements on each study participant. A 2-sided *P* value <.05 was considered statistically significant. No adjustment for multiple comparison was performed. Graphs were made with GraphPad Prism 9.

## Results

### Baseline Characteristics

In the first part of the study, 15 men with T1D and 15 healthy controls were included. There were no differences between groups in age, sex, and body mass index (Table 1). However, the mean fasting PG and hemoglobin A1c were significantly higher in the group with T1D compared with the controls. None of the participants had any history of coagulopathies or were on any anticoagulant or antiplatelet treatment. In the second part of the study, the same people with T1D as participated in the first part were included, and further details of their baseline characteristics have previously been reported (18).

**Table 1.** Baseline characteristics of participants with T1D and healthy controls

	T1D (N = 15)	Controls (N = 15)	*P*
Age (y)	29 ± 8	30 ± 8	.863
Male N (%)	15 (100)	15 (100)	1.000
BMI (kg/m^2^)	23.7 ± 2.0	23.9 ± 2.2	.859
HbA_1c_ (%)	6.8 ± 0.5	5.4 ± 0.3	<.001
HbA_1c_ (mmol/mol)	51.0 ± 5.5	32.0 ± 1.9	<.001
FPG (mmol/L)	9.8 ± 2.0	5.5 ± 0.3	<.001
Hemoglobin (mmol/L)	9.6 ± 0.5	9.4 ± 0.6	.442
INR	1.1 ± 0.1	1.1 ± 0.1	.564
Platelet count (×10^9^ L)	244 ± 57	223 ± 52	.295
Systolic blood pressure (mm Hg)	125 ± 11	121 ± 11	.280
Diastolic blood pressure (mm Hg)	77 ± 12	77 ± 7	.880
Heart rate (beats/min)	65 ± 11	62 ± 9	.385

Categorical data are presented as N (%), and continuous variables are presented as mean ± SD. Further details of baseline characteristics of the participants with type 1 diabetes have been described elsewhere (18).

Abbreviations: BMI, body mass index; FPG, fasting plasma glucose; HbA_1c_, glycated hemoglobin A_1c_; INR, international normalized ratio; T1D, type 1 diabetes.

### Baseline Hemostatic Comparison Between Groups

Individuals with T1D had a shorter R-time and K-time compared with the controls, as well as a greater α-Angle (Table 2). A numerically higher MA was observed for people with T1D compared with the controls; however, the result was not statistically significant. No differences were found in MA-FF or LY-30 between groups.

**Table 2.** Comparison of hemostatic profiles between individuals with T1D and matched controls

TEG variable	Group	Mean	95% CI	*P*
R-time (min)	T1D	5.41	4.92-5.90	
	Controls	7.03	6.37-7.69	
	Difference	−1.62	−2.41 to −0.84	<.001
K-time (min) †	T1D	1.44	1.35-1.54	
	Controls	1.77	1.51-2.07	
	Ratio (T1D/controls)	0.81	0.69-0.96	.019
α-Angle (**°**)	T1D	71.7	70.9-72.4	
	Controls	68.6	66.5-70;7	
	Difference	3.0	0.8-5.3	.010
MA (mm)	T1D	56.5	54.8-58.2	
	Controls	53.7	50.7-56.7	
	Difference	2.8	−0.5 to 6.2	.090
MA-FF (mm)	T1D	16.1	15.1-17.2	
	Controls	15.8	14.6-17.1	
	Difference	0.3	−1.2 to 1.8	.691
LY-30 (%)	T1D	1.12	0.55-1.69	
	Controls	0.97	0.56-1.39	
	Difference	0.15	−0.53 to 0.83	.651

Data are presented as mean with 95% CIs. Data with non-normal distributions were logarithmically transformed before statistical comparison and are marked with (†).

Abbreviations: Ly-30, measurement of fibrinolysis; MA, maximum amplitude; MA-FF, maximum amplitude of functional fibrinogen; T1D, type 1 diabetes; TEG, thrombelastography.

### Clamped Plasma Glucose

A detailed overview of PG on both test days has been reported elsewhere (18). At baseline, the PG (mean ± SD) was 6.1 ± 1.1 mmol/L and 5.9 ± 0.6 mmol/L on Hypo-exercise and Hypo-rest, respectively. After 15 minutes of hypoglycemia, the mean PG was 2.7 ± 0.3 mmol/L on Hypo-exercise and 2.6 ± 0.3 mmol/L on Hypo-rest. After 60 minutes of hypoglycemia, the PG was 2.5 ± 0.3 mmol/L and 2.6 ± 0.1 mmol/L, on Hypo-exercise and Hypo-rest, respectively. After 30 minutes of recovery, the PG was 6.8 ± 1.2 mmol/L on Hypo-exercise and 6.2 ± 1.3 mmol/L on Hypo-rest, whereas, after 24 hours of recovery, the mean PG was 10.4 ± 3.2 mmol/L and 10.8 ± 3.0 mmol/L on Hypo-exercise and Hypo-rest, respectively. No differences in PG level were observed at any study time point. There was no difference in the amount of insulin infused between the test days. However, as previously reported (18), the amount of glucose infused was higher on Hypo-exercise compared with Hypo-rest.

### Plasma Adrenaline and Noradrenaline

The baseline plasma adrenaline level was similar on both test days. After 15 minutes of hypoglycemia, the adrenaline level increased from baseline on both test days however, the increase was greater on Hypo-exercise compared with Hypo-rest (Table 3). After 60 minutes of hypoglycemia, the adrenaline level remained increased from baseline on both test days. At this point, the increase in Δadrenaline was numerically higher on Hypo-rest compared with Hypo-exercise but not statistically significant. After 30 minutes of recovery, adrenaline remained increased from baseline on both test days and with no differences between the test days. Plasma noradrenaline increased from baseline after 15 minutes of hypoglycemia on Hypo-exercise and remained increased after 60 minutes of hypoglycemia and after 30 minutes of recovery (Table 3). On Hypo-rest, the plasma noradrenaline increased from baseline after 60 minutes of hypoglycemia and return to baseline levels after 30 minutes of recovery. Further details are presented in Table 3.

**Table 3.** Changes in plasma adrenaline, noradrenaline, and markers of endothelial activation and damage during hypoglycemia and recovery

Outcome	Test day	Baseline		Hypoglycemia (15 min)			Hypoglycemia (60 min)			Recovery (30 min)			Recovery (24 h)		
				(ref. baseline)			(ref. baseline)			(ref. baseline)			(ref. baseline)		
		Mean	95% CI	ΔMean	95% CI	*P*	ΔMean	95% CI	*P*	ΔMean	95% CI	*P*	ΔMean	95% CI	*P*
Adrenaline (ng/mL) †	Hypo-exercise	0.02	0.01-0.03	14.4	9.3-22.2	<.001	6.4	4.3-9.5	<.001	1.9	1.29-2.67	.003	NA	NA	NA
	Hypo-rest	0.02	0.01-0.03	5.1	2.5-10.4	<.001	10.7	5.9-19.3	<.001	2.2	1.46-3.15	.001	NA	NA	NA
	Hypo-exercise vs Hypo-rest			2.8	1.5-5.2	.003	0.6	0.3-1.2	.123	0.9	0.51-1.46	.557	NA	NA	NA
Noradrenaline (ng/mL)	Hypo-exercise	0.19	0.14-0.25	0.50	0.25-0.76	<.001	0.07	0.03-0.11	.001	0.08	0.01-0.15	.026	NA	NA	NA
	Hypo-rest	0.19	0.13-0.26	0.03	−0.01-0.07	.088	0.06	0.02-0.10	.008	0.02	−0.03 to 0.07	.251	NA	NA	NA
	Hypo-exercise vs Hypo-rest			0.47	0.22-0.73	.002	0.01	−0-03 to .05	.492	0.06	−0.02 to 0.13	.111	NA	NA	NA
Thrombomodulin (ng/mL) †	Hypo-exercise	2.4	1.7-3.0	1.0	0.9-1.2	.672	0.9	0.8-1.1	.152	0.9	0.7-1.0	.122	1.1	1.0-1.2	.174
	Hypo-rest	1.7	1.5-1.9	1.0	1.0-1.1	.363	1.0	0.9-1.1	.864	0.9	0.8-1.0	.004	1.1	0.9-1.2	.350
	Hypo-exercise vs Hypo-rest			1.0	0.9-1.2	.461	0.9	0.7-1.1	.194	1.0	0.8-1.2	.957	1.0	0.9-1.20	.740
Syndecan-1 (ng/mL) †	Hypo-exercise	48.1	15.6-80.6	1.0	0.9-1.1	.857	0.9	0.8-1.0	.056	0.9	0.8-1.1	.407	1.0	0.9-1.2	.830
	Hypo-rest	40.1	17.6-62.5	1.1	1.0-1.2	.134	1.1	1.0-1.2	.110	0.9	0.8-1.0	.146	1.0	0.9-1.1	.829
	Hypo-exercise vs Hypo-rest			0.9	0.8-1.0	.167	0.8	0.7-0.9	.004	1.0	0.9-1.2	.802	1.0	0.9-1.1	.970
PECAM-1 (ng/mL)	Hypo-exercise	7.0	5.2-8.7	0.8	0.1-1.5	.035	−0.2	−0.9 to 0.5	.562	−0.5	−1.1 to 0.0	.055	0.6	−0.3 to 1.4	.184
	Hypo-rest	8.4	7.1-9.8	−0.1	−1.0-0.7	.737	−0.2	−1.6 to 1.1	.717	−0.9	−2.1 to 0.4	.160	0.1	−1.2 to 1.3	.936
	Hypo-exercise vs Hypo-rest			0.9	−0.4-2.3	.158	0.0	−1.7-1.8	.964	0.4	−1.1 to 1.8	.623	0.5	−1.2 to 2.3	.533

Data with non-normal distributions were logarithmically transformed before statistical comparison and are marked with (†). For data with normal distribution, Δmean is represented by differences within or between test days of arithmetic means, whereas for logarithmically transformed data, Δmean is the ratio of geometric means within or between test days.

Abbreviation: ref., reference.

### Rate of Coagulation Activation During Hypoglycemia and Recovery

**Figure 2.**
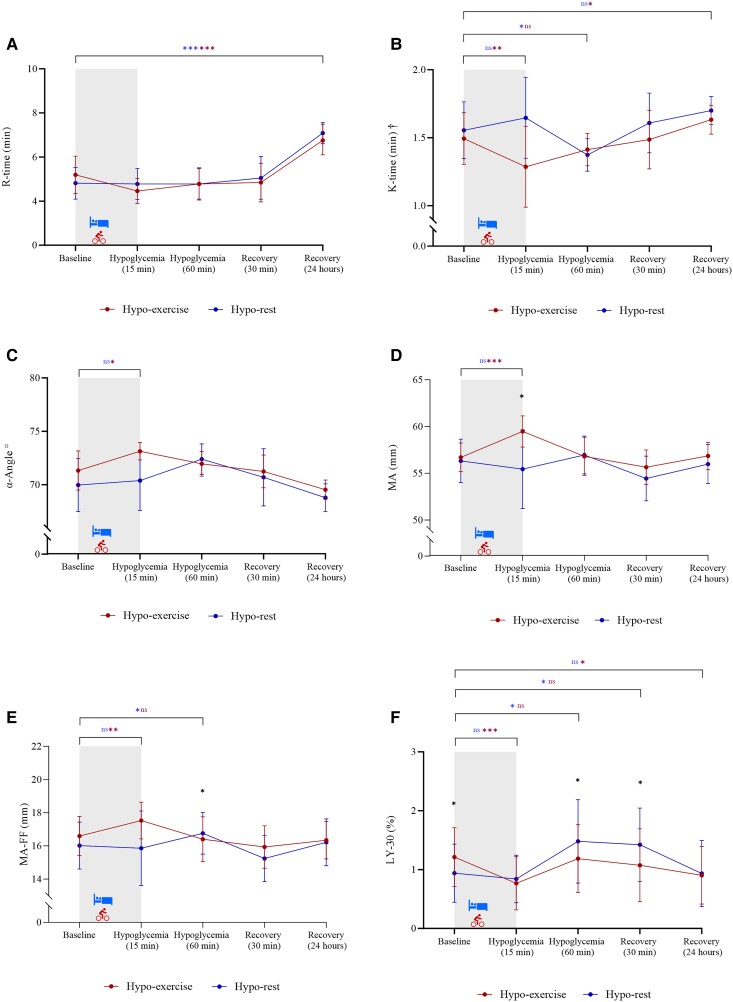
Changes in TEG parameters during and after exercise-related hypoglycemia vs hypoglycemia at rest in individuals with T1D. Continuous data are presented as mean with 95% CI. Data with non-normal distributions were logarithmically transformed before statistical comparisons and are marked with (†). Hypo-exercise is colored in red, whereas Hypo-rest is colored in blue. Blue or red colored asterisk represents significant difference from baseline within the same test day, whereas black colored asterisk represents significant difference of the delta values between test days.

Abbreviations: MA, maximum amplitude; min, minutes; MA-FF, maximum amplitude of functional fibrinogen; ns, nonsignificant; T1D, type 1 diabetes; TEG, thrombelastography. ns, *P* > .05; *P < .05; **P < .01; ***P < .001.

Compared with baseline, no changes in R-time were observed during hypoglycemia or same-day recovery. However, after 24 hours of recovery, the R-time increased from baseline on both test days. No differences in ΔR-time were observed between test days at any time points (Fig. 2A, Supplementary Table S2 (23)).

### Rate of Clot Formation During Hypoglycemia and Recovery

After 15 minutes of hypoglycemia, a decrease in K-time from baseline was only observed on Hypo-exercise but with no difference between test days. On the contrary, after 60 minutes of hypoglycemia, a decrease in K-time from baseline was only observed on Hypo-rest but there was no difference between test days. After 24 hours of recovery, an increase in K-time was observed only on Hypo-exercise but with no difference between test days (Fig. 2B, Supplementary Table S2 (23)). After 15 minutes of hypoglycemia an increase in α-Angle from baseline was observed only on Hypo-exercise, without any difference between test days observed. After 60 minutes of hypoglycemia, a numerical but not significant increase in α-Angle was observed only on Hypo-rest and the increase was greater compared with Hypo-exercise (Fig. 2C, Supplementary Table S2 (23)). No changes were observed within or between test days during recovery from hypoglycemia.

### Clot Strength

After 15 minutes of hypoglycemia an increase in MA from baseline was observed only on Hypo-exercise. The increase in ΔMA on Hypo-exercise was higher compared with Hypo-rest (Fig. 2D, Supplementary Table S2 (23)). For the remaining time points, no changes in MA were observed within or between test days.

### Functional Fibrinogen Contribution to Clot Strength

After 15 minutes of hypoglycemia, the MA-FF increased from baseline only on Hypo-exercise but with no observed difference between test days. In contrast, after 60 minutes of hypoglycemia an increase in ΔMA-FF was only observed on Hypo-rest. The increase in ΔMA-FF was higher compared with Hypo-exercise (Fig. 2E, Supplementary Table S2 (23)).

### Fibrinolysis

After 15 minutes of hypoglycemia a decrease in LY-30 from baseline was observed on both test days; however, the result was only significant on Hypo-exercise (Fig. 2F, Supplementary Table S2 (23)). No difference in ΔLY-30 between test days was observed at this time point. After 60 minutes of hypoglycemia and 30 minutes of recovery the LY-30 was similar to baseline on Hypo-exercise but increased on Hypo-rest. The increase in ΔLY-30 on Hypo-rest was higher compared with Hypo-exercise during both these time points. After 24 hours of recovery, the LY-30 was decreased compared to baseline only on Hypo-exercise and with no difference between test days observed.

### Endothelial Activation and Damage

Thrombomodulin showed no significant changes from baseline during hypoglycemia on either of the test days; however, a decrease from baseline was observed after 30 minutes of recovery on Hypo-rest only (Table 3). An increase in PECAM-1 from baseline was observed after 15 minutes on Hypo-exercise, but with no differences between the test days (Table 3). No significant changes in the level of syndecan-1 were observed during the study (Table 3).

### Vascular Oxidative Stress

At baseline, there was no difference between test days in plasma levels of L-arginine, ADMA, L-arginine/ADMA ratio, or malondialdehyde. Both the L-arginine and L-arginine/ADMA ratio decreased after 15 and 60 minutes of hypoglycemia and after 30 minutes of recovery on both test days. No differences in delta values were observed for any of these markers between the test days (Supplementary Table S1 (23)). At baseline, the concentration of the total ascorbic acid was similar between test days. The concentration remained unchanged from baseline throughout both test days (Supplementary Fig. S1 (23)). However, both the baseline levels of DHAA and %DHAA were higher on Hypo-exercise compared with Hypo-rest (Supplementary Fig. S1, Supplementary Table S1 (23)). On Hypo-exercise, a decrease in %DHAA from baseline was observed after 15 minutes of hypoglycemia, whereas on Hypo-rest an increase in %DHAA from baseline was observed after 60 minutes of hypoglycemia (Supplementary Fig. S1 (23)) The Δ%DHAA was higher on Hypo-rest compared to Hypo-exercise during all time points.

## Discussion

In the present study, we used a whole-blood viscoelastic test to compare hemostatic profiles of young men with T1D with matched healthy controls and to delineate acute hemostatic effects of exercise-related hypoglycemia in comparison with hypoglycemia induced at rest. Our main findings are that (1) young men with T1D had a hypercoagulable profile compared with healthy controls, (2) exercise-related hypoglycemia was associated with increased clot strength together with a decreased fibrinolytic activity, and (3) hypoglycemia induced at rest was associated with an increased contribution of functional fibrinogen to the clot strength along with increased fibrinolytic activity.

The differences observed in hemostatic profiles between young men with T1D and the healthy controls were primarily related to the rate of coagulation activation and the rate of clot formation, including the polymerization process of functional fibrinogen. Our findings are to some extent in accordance with a previous study conducted in a pediatric population, using rotational thromboelastometry (8). The authors showed that children with T1D had a faster rate of coagulation activation and a higher maximum clot strength compared with the controls. In the present study, the clot strength was numerically but not statistically higher in the group with T1D, which may be due to a lack of power. The underlying cause of a more hypercoagulable hemostatic profile in individuals with T1D remain unclear. However, we speculate that the hyperglycemic state of T1D can, at least partly, explain our findings because it has previously been shown that hyperglycemia associates with procoagulant effects, including increased levels of circulating tissue factor, prothrombin fragment 1.2, and factor VIII (21, 22). Our results add to the current literature, suggesting that young otherwise healthy men with T1D may have a lower threshold for coagulation activation. Nevertheless, the present findings need to be confirmed by others and future studies are needed to evaluate the clinical implications, including the risk of thrombosis, of the TEG 6s-assessed hypercoagulable state of T1D.

To our knowledge, this is the first study to delineate acute hemostatic effects of exercise-related hypoglycemia and hypoglycemia induced at rest in individuals with T1D. To explore the dynamics in hemostasis over time, TEG was performed both after 15 and 60 minutes’ duration of hypoglycemia. We show that the combination of exercise and hypoglycemia can induce a shift in hemostasis toward a more prothrombotic state, including procoagulant and antifibrinolytic effects. Previous studies investigating the hemostatic effects of acute exercise in healthy individuals under euglycemic circumstances have shown that the hemostatic balance can be maintained by both increased coagulation and fibrinolysis (24, 25). Interestingly, Weiss et al showed that moderate-intensity exercise was associated with enhanced fibrinolysis without concomitant activation of coagulation (25). Noteworthily, individuals with T1D have been shown to have an intact fibrinolytic potential during exercise (26, 27). In the present study, a numerical but not significant decrease in fibrinolytic activity was observed after 15 minutes of hypoglycemia induced at rest; thus, whether the impaired fibrinolysis observed after 15 minutes of exercise-related hypoglycemia is a result of exercise, hypoglycemia, or a combination of both remains uncertain. It might be speculated that the decreased fibrinolysis was a result of changes in the fibrin clot structure with increased resistance to clot breakdown (28). A striking observation was that the hypercoagulable changes observed after 15 minutes of exercise-related hypoglycemia were resolved after 60 minutes of hypoglycemia. This may be explained by a blunted sympathoadrenal response because same-day exercise has previously been shown to diminish the adrenaline response to subsequent hypoglycemia in previous studies (29, 30). This theory is further supported by the fact that when hypoglycemia was induced at rest, the hypercoagulable changes were first observed after 60 minutes of hypoglycemia and were accompanied by a peak increase in adrenaline. Accordingly, similar temporal differences were observed for plasma noradrenaline, where a peak increase was observed after 15 minutes of exercise-related hypoglycemia and after 60 minutes of hypoglycemia induced at rest, respectively. Given that both adrenaline and noradrenaline previously have been associated with acute prothrombotic effects, including platelet hyperreactivity and coagulation activation (31, 32), it is likely that the hemostatic effects observed in the present study, were mediated by these hormones.

To delineate persistency in the hemostatic effects observed during hypoglycemia, TEG was performed during recovery the same day and after 24 hours. We observed that only exercise-related hypoglycemia was associated with a decreased fibrinolytic activity after 24 hours of recovery, potentially prolonging the thrombotic susceptibility. In a study performed by Chow et al in a type 2 diabetes population, hypoglycemia induced at rest was associated with an impaired fibrinolysis both after 24 hours and 7 days of recovery (15). Surprisingly, and for unknown reasons, both test days were associated with anticoagulant changes, as measured by an increased R-time after 24 hours of recovery. Given that the R-time was unchanged during hypoglycemia on both test days and that administration of insulin previously has been associated with anti-inflammatory effects, we speculate that the hyperinsulinemic clamp technique may have contributed to these anticoagulant effects after 24 hours (33).

We also explored the acute effects of hypoglycemia on endothelial function and vascular oxidative stress, both of which have been reported as important factors in the development of atherosclerosis (34, 35). Overall, we observed sparse effects of hypoglycemia on these outcomes. However, we did observe a reduction in thrombomodulin during same-day recovery from hypoglycemia only when induced under resting conditions. Thrombomodulin is a glycoprotein located on the surface of the endothelial cells and plays an important role in anticoagulation by activating protein C (36). Thus, our data indicate transient procoagulant effects of the endothelium beyond the hypoglycemic event. This is in line with a previous study, showing that antecedent hypoglycemia can result in endothelial dysfunction and a prothrombotic state in individuals with T1D (13). Nevertheless, because no similar changes were observed during hypoglycemia in the present study, we cannot exclude the possibility of a spurious finding. Finally, we used the oxidation product of ascorbic acid (%DHAA) as a marker of oxidative stress (21). Interestingly, we observed that hypoglycemia induced at rest was associated with increased oxidative stress, whereas exercise-related hypoglycemia was not. However, despite the randomized design of the present study, the baseline levels of %DHAA were not similar on the 2 test days, which makes these results difficult to interpret.

The present study has some limitations. First, the small sample size of this study may have increased the risk of type II errors. However, a crossover design was used to improve statistical power. Second, the participants of this study were young males with no history of cardiovascular disease before inclusion. The inclusion of only 1 sex and a narrow age range of the participants diminish generalizability and may increase the risk of biased interpretation of the results with respect to T1D in general. However, the decision not to include older individuals was due to ethical and safety reasons because hypoglycemia transiently induces cardiovascular stress. Similarly, only including males in the present study increased statistical power because previous studies have reported cyclic variations in hemostasis in premenopausal women, which may cause confounding results because of period effects (37). Third, because the exploratory nature of this study and the use of unadjusted *P* values, the results of this study are indicative; hypothesis-generating rather than conclusive and spurious findings (type I error) cannot be ruled out.

In conclusion, we show that young male individuals with T1D have a hypercoagulable profile compared with matched healthy controls, potentially alluding to the increased susceptibility to thrombotic events associated with T1D. Furthermore, we show that exercise-related hypoglycemia induces a shift in the hemostatic balance through procoagulant and antifibrinolytic effects, of which the latter was present 24 hours after recovery from hypoglycemia. In contrast, a more maintained hemostatic balance was observed when hypoglycemia was induced at rest. Taken together, our findings suggest that the underlying physical circumstances in relation to hypoglycemia (exercise vs rest) may be of clinical importance for hypoglycemia-induced disturbances in coagulability. Further studies are needed to confirm our findings and to investigate changes in hemostasis during different intensity levels of exercise at different glycemic levels in people with T1D.

## Data Availability

Because of Danish data protection laws, the datasets generated and analyzed during the current study are not publicly available but are available from the corresponding author on reasonable request.

## Clinical Trial Registration Number

NCT04650646.

## Prior Presentation

This study was presented at The American Diabetes Association 82nd Scientific Sessions, June 3-7, 2022, and at the 58th Annual Meeting of the European Association for the Study of Diabetes, September 19-23, 2022.

## Abbreviations

ADMA: asymmetric dimethylarginine
DHAA: dehydroascorbic acid
K-time: rate of clot formation
LY-30: fibrinolysis
MA: maximum clot amplitude
MA-FF: functional fibrinogen contribution to the clot strength
PECAM-1: platelet endothelial cell adhesion molecule 1
PG: plasma glucose
R-time: reaction time
T1D: type 1 diabetes
TEG: thrombelastography
